# EU Global Health Strategy: what are the challenges?

**DOI:** 10.1093/eurpub/ckad081

**Published:** 2023-06-06

**Authors:** Nina Viberg, Rhoda Wanyenze, Helena Nordenstedt, Githinji Gitahi, Stefan Swartling Peterson

**Affiliations:** Global Public Health Sciences, Karolinska Institutet, Solna, Sweden; College of Health Sciences, School of Public Health, Makerere University, Uganda; Global Public Health Sciences, Karolinska Institutet, Solna, Sweden; AMREF, Kenya; Global Public Health Sciences, Karolinska Institutet, Solna, Sweden; College of Health Sciences, School of Public Health, Makerere University, Uganda

Last November the European Commission presented its new forward-looking Global Health Strategy.^1^ The strategy provides an agenda up to year 2030 that outlines what the Commission will do and what it invites Member States to do. It sets three policy priorities, namely to: (i) deliver better health and well-being of people across the life course; (ii) strengthen health systems and advance universal health coverage; and (iii) prevent and combat health threats, including pandemics, applying a One Health approach. Furthermore, the Strategy puts forward twenty guiding principles to shape global health and then makes concrete lines of action for operationalization of the principles. To assess progress and ensure the accountability of the EU’s global health action, permanent monitoring and assessment will be set up.

The Strategy positions global health as an essential pillar of EU external policy and promotes sustainable, meaningful partnerships of equals. Described as the external dimension of the European Health Union, the strategy is designed to guide EU action for ensuring better preparedness and response to health threats in a seamless way. Further, the strategy seeks to leverage a Team Europe Approach,^1^ initially put in place to ensure a coordinated and comprehensive response between the EU and its Member States to the COVID-19 pandemic and its consequences. The EU Global Health Strategy also introduces a robust ‘health-in-all-policies’ approach to ensure that a wide variety of policies genuinely contribute to health goals. It identifies three key enablers for better health, namely digitalization, research and a skilled labour force.

As part of the Swedish presidency of the Council of Europe, the Karolinska Institutet (KI) arranged a seminar to contribute to the operalization of the strategy. Global health experts and stakeholders from Europe as well as other continents EU participated, representing academia, civil society organizations, policymakers and private sector. KI president Ole Petter Ottersen, previous chair on the Lancet-University of Oslo Commission on Global Governance for Health^2^ called on the event to be a platform based on voices of all relevant stakeholders from all disciplines. ‘Health should be dealt with from a global perspective. Health is by nature global’.

While it is laudable that EU now has a global health strategy, what are the challenges, and how can it be operationalized? These were the core issues up for debate on the seminar.

- Is a global health strategy a specific EU responsibility? Whether from a decolonization^3^ or health determinants^4^ perspective it can be argued that all parts of the world should have a Global Health Strategy, so maybe one should agree on forming a Global Health Strategy—EU, a Global Health Strategy—AU (African Union), etc. Is the EU Global Health Strategy a health security strategy rather than a holistic healthy populations strategy? A strong focus on infectious diseases that risk spreading to the EU might divert the much-needed attention on health issues such as non-communicable diseases, where EU exports of e.g. non-health promoting foods are not compatible with a ‘health-in-all-policies’ approach, as per the strategy.
- The implementation of the EU strategy is vested in the EU institutions and member states—in itself an ambitious undertaking but essentially more of a whole-of-government approach than whole-of-society one.^5^ Where are the meeting places, and ‘horizontal governance’ mechanisms that will enable the crucial multi-sectoral and trans-disciplinary work including actors such as civil society, academia and private sector? A meeting a year in the Global Health Policy Forum is commendable but will not be enough.
- Ultimately Global Health is developed and delivered locally, in communities, and the Global therefore needs to become ‘hyperlocal’. Achieving the strategy’s objectives will therefore depend on bottom-up and local considerations.
- The seminar also brought to the table some of the drawbacks that the rest of the world experienced from the joint EU covid response and argued that implementation of the EU Global Health strategy must build on the learnings from that and strive towards a more, attentive, inclusive and equitable approach.

Just as the ‘European Coal and Steel Community’ once introduced a common system to integrate sectors across national borders, the challenge now is to, while attentively and inclusively interacting with the rest of the world, operationalize the EU Global Health Strategy to create integrated and coordinated ‘Systems for One Health’ across sectors and actors aligning the Green Deal to synergize for healthy populations as well as better health security worldwide.

*Conflicts of interest*: None declared.

## References

[1] EU Global Health Strategy. 2014. Available at: europa.eu (29 April 2023, date last accessed).

[2] The Lancet—University of Oslo Commission on Global Governance for Health (29 April 2023, date last accessed).

[3] Patterson D , Woldeyes M, Schuck N, et al Decolonising global health in Africa: research agendas in public health, law, and human rights. Eur J Public Health 2022;32:ckac131.100.

[4] Commercial determinants of health. Available at: https://www.thelancet.com/ (29 April 2023, date last accessed).

[5] Kickbusch I , Gleicher D. *Governance for Health in the 21st Century*. World Health Organization. Regional Office for Europe, 2012. Available at: https://apps.who.int/iris/handle/10665/326429.

